# Maternal tadalafil treatment does not increase uterine artery blood flow or oxygen delivery in the pregnant ewe

**DOI:** 10.1113/EP091593

**Published:** 2024-04-12

**Authors:** Jack R. T. Darby, Dimitra Flouri, Steven K. S. Cho, Georgia K. Williams, Stacey L. Holman, Ashley S. Meakin, Michael D. Wiese, Anna L. David, Christopher K. Macgowan, Mike Seed, Andrew Melbourne, Janna L. Morrison

**Affiliations:** ^1^ Early Origins of Adult Health Research Group, Health and Biomedical Innovation, UniSA: Clinical and Health Sciences University of South Australia Adelaide South Australia Australia; ^2^ School of Biomedical Engineering and Imaging Sciences King's College London London UK; ^3^ Univeristy of Toronto and The Hospital for Sick Children Toronto Ontario Canada; ^4^ Preclinical, Imaging & Research Laboratories South Australian Health & Medical Research Institute Adelaide Australia; ^5^ Centre for Pharmaceutical Innovation, UniSA: Clinical and Health Sciences University of South Australia Adelaide South Australia Australia; ^6^ Elizabeth Garrett Anderson Institute for Women's Health University College London London UK; ^7^ National Institute for Health and Care Research (NIHR) University College London, Hospitals Biomedical Research Centre London UK

**Keywords:** fetal development, fetal growth restriction, fetal MRI, haemodynamics, magnetic resonance imaging, placental perfusion, tadafer, tadalafil

## Abstract

Increasing placental perfusion (PP) could improve outcomes of growth‐restricted fetuses. One way of increasing PP may be by using phosphodiesterase (PDE)‐5 inhibitors, which induce vasodilatation of vascular beds. We used a combination of clinically relevant magnetic resonance imaging (MRI) techniques to characterize the impact that tadalafil infusion has on maternal, placental and fetal circulations. At 116–117 days’ gestational age (dGA; term, 150 days), pregnant ewes (*n* = 6) underwent fetal catheterization surgery. At 120–123 dGA ewes were anaesthetized and MRI scans were performed during three acquisition windows: a basal state and then ∼15–75 min (TAD 1) and ∼75–135 min (TAD 2) post maternal administration (24 mg; intravenous bolus) of tadalafil. Phase contrast MRI and T_2_ oximetry were used to measure blood flow and oxygen delivery. Placental diffusion and PP were assessed using the Diffusion‐Relaxation Combined Imaging for Detailed Placental Evaluation—‘DECIDE’ technique. Uterine artery (UtA) blood flow when normalized to maternal left ventricular cardiac output (LVCO) was reduced in both TAD periods. DECIDE imaging found no impact of tadalafil on placental diffusivity or fetoplacental blood volume fraction. Maternal‐placental blood volume fraction was increased in the TAD 2 period. Fetal $D_{O_{2}}$ and ${\dot{V}}_{O_{2}}$ were not affected by maternal tadalafil administration. Maternal tadalafil administration did not increase UtA blood flow and thus may not be an effective vasodilator at the level of the UtAs. The increased maternal–placental blood volume fraction may indicate local vasodilatation of the maternal intervillous space, which may have compensated for the reduced proportion of UtA $D_{O_{2}}$.

## Abstract (highlights)

- **What is the central question of this study?**
  Tadalafil is under consideration as an intervention strategy for fetal growth restriction: what is the impact of tadalafil on uterine artery blood flow, placental perfusion and fetal oxygen delivery?
- **What is the main finding and its importance?**
  Tadalafil does not increase uterine artery blood flow, placental perfusion or fetal oxygen delivery. Tadalafil may not be suitable as an intervention strategy for fetal growth restriction. The MRI techniques used herein would aid in the appropriate selection of future intervention strategies.

## 1 INTRODUCTION

In humans, fetal growth restriction (FGR) is associated with reduced uterine artery (UtA) blood flow and impaired placental perfusion (PP) (Ferrazzi et al., 2011; Konje et al., 2003; Liu et al., 2022). This increases the risk of poor in utero, neonatal and long‐term outcomes (Bukowski et al., 2014; Gardosi et al., 2005; Sasi et al., 2015). Given that there are currently no proven treatments to prevent FGR or improve associated poor outcomes in utero, the current clinical guideline is to optimize the timing of delivery in order to mitigate the risk of stillbirth (Zeitlin et al., 2000). Development of an intervention strategy capable of increasing PP and fetal substrate transport, thereby delaying delivery after detection of FGR, would significantly improve short‐ and long‐term health outcomes in this population.

In recent years, phosphodiesterase‐5 (PDE_5_) inhibitors, which are capable of inducing vasodilatation of vascular beds, have been a hot topic within the fetal and perinatal research community (Darby, 2020; Inocencio et al., 2020). One of these inhibitors, sildenafil, was assessed in the Sildenafil TheRapy in Dismal Prognosis Early‐onset fetal Growth Restriction (STRIDER) trials but found to be ineffective and potentially harmful (Sharp et al., 2018; Groom et al., 2019; Pels et al., 2020). As a consequence of these poor outcomes, a lesser‐known concurrent phase II trial investigating the use of an alternate PDE‐5 inhibitor, tadalafil, was asked by their funding agency to cease recruitment (Umekawa et al., 2018). This Safety Evaluation of Tadalafil Treatment for Fetuses with Early‐Onset Growth Restriction (TADAFER) phase II trial was built on the premise that tadalafil not only has a longer half‐life than sildenafil (14–15 vs. 2–4 h), which would improve stability and potentially effectiveness (Park et al., 2018), but also on the increased selectivity that tadalafil has toward the reproductive organs (Wright, 2006). Leading up to the TADAFER phase II trial, small case studies found that not only was UtA blood flow correlated with maternal tadalafil concentrations (Tanaka et al., 2019), but when assessed retrospectively, oral maternal tadalafil treatment (20 mg per day) appeared to increase birthweight and growth velocity of the growth‐restricted fetus (Kubo, Umekawa et al., 2017). Whilst the TADAFER phase II trial did not complete full recruitment (89/140 FGR fetuses enrolled; Umekawa et al., 2018), results were analysed for safety considerations. Encouragingly, albeit in an incomplete dataset, oral maternal tadalafil treatment (20 mg per day) was associated with significantly decreased fetal, neonatal and infant death (Maki et al., 2019). This was attributable to a significantly later gestational age (GA) at delivery for women in the tadalafil intervention group compared to those in the placebo group. This positive finding has since led to approval and commencement of recruitment for a further ‘exploratory’ phase II trial, namely TADAFER IIb, in which the efficacy of multiple doses per day will be investigated (Maki et al., 2022).

Given the negative results and safety concerns observed with sildenafil, it is imperative that we fully understand the impact that such interventions have on the maternal, placental and fetal circulations. Previously, we have used preclinical sheep and human studies to validate the use of phase contrast (PC) magnetic resonance imaging (MRI) and T_2_ oximetry to calculate oxygen delivery within the maternal and fetal circulations and applied these techniques to characterize the impact of vasoactive agents on fetal haemodynamics (Darby et al., 2020; Duan et al., 2019; Morrison et al., 2022; Saini et al., 2021, 2020). More recently, we have targeted the placenta itself by developing a multicompartment model of sheep placental tissue to allow for the assessment of placental blood flow and permeability (Diffusion‐rElaxation Combined Imaging for Detailed Placental Evaluation; DECIDE; Flouri et al., 2021). Importantly, not only is this model capable of detecting low placental $S_{O_{2}}$ and thereby distinguishing small hypoxaemic fetuses from normoxaemic appropriately grown fetuses, but when translated to humans it is able to identify differences in placental $S_{O_{2}}$ between growth‐restricted fetuses with and without normal Doppler flows (Aughwane et al., 2021; Flouri et al., 2022). This highlights the translatable capacity of advanced MRI imaging and suggests that these techniques may prove useful in assessing the efficacy of interventions against FGR.

Herein, we aimed to utilize a combination of the clinically relevant PC‐MRI and T_2_ oximetry and DECIDE imaging to determine the impact of tadalafil on maternal haemodynamics, PP and fetal oxygen delivery ($D_{O_{2}}$). We hypothesized that tadalafil would increase UtA blood flow leading to an increase in fetal oxygen delivery, measurable both at the placenta and within the fetal circulation.

## 2 METHODS

### 2.1 Ethical approval

All experimental protocols were reviewed and approved by the Animal Ethics Committee of the South Australian Health and Medical Research Institute (SAHMRI) and abide by the Australian Code of Practice for the Care and Use of Animals for Scientific Purposes developed by the National Health and Medical Research Council. Ewes from the SAHMRI farm (Burra, South Australia) were housed in an indoor facility with a constant ambient temperature of 20–22°C and a 12‐h light–dark cycle. Ewes were housed in individual pens in view of other sheep and had ad libitum access to food and water. All investigators understood the ethical principles of the journal (Grundy, 2015) and the principles of the 3Rs, specifically the reduction of the use of animals in research (Russell & Burch, 1959).

### 2.2 Fetal catheterization surgery

At 116–117 days GA (dGA), Merino ewes (*n* = 7; male; female, 4:3) underwent surgery as previously described (Darby et al., 2018; Morrison et al., 2001). Anaesthesia was induced with intravenous diazepam (0.3 mg/kg) and ketamine (5 mg/kg) and then maintained with isoflurane (1.5–2.5% in 100% oxygen). Vascular catheters were implanted into the maternal jugular vein, fetal femoral vein, femoral artery and the amniotic cavity as previously described (Morrison et al., 2001). Ewes received an intramuscular injection of antibiotics (3.5 mL of Duplocillin; 150 mg/mL procaine penicillin and 112.5 mg/mL benzathine penicillin; Norbrook Laboratories Ltd, Gisborne, Australia) and 2 mL of 125 mg/mL dihydrostreptomycin (Sigma‐Aldrich, St Louis, MO, USA) at surgery and for 3 days following surgery. Fetuses received an intramuscular injection of 1 mL of Duplocillin (150 mg/mL procaine penicillin and 112.5 mg/mL benzathine penicillin); and 1 mL of 125 mg/mL Dihydrostreptomycin during surgery. All ewes received an analgesic, meloxicam (0.5 mg/kg, subcutaneously) on the day before surgery and 24 h later (Varcoe et al., 2019). Each fetus received antibiotics (500 mg; sodium ampicillin, Commonwealth Serum Laboratories, Melbourne, Australia) intra‐amniotically for 4 days post‐surgery.

### 2.3 Experimental protocol

Pregnant ewes (61.1 ± 5.6 kg; mean ± SD) underwent MRI scans between 120 and 124 dGA after 16 h of fasting. General anaesthesia was induced in the ewe as described for surgery above. A temporary fluid‐filled arterial line was then placed in the forelimb of the ewe. The ewe was then positioned on its left side for the duration of the scan and ventilated to ensure normal fetal oxygenation levels (respiratory rate 16–18; ∼1 L O_2_ and 5 L air). Maternal heart rate and arterial oxygen saturation were measured using an MRI‐compatible $S_{aO_{2}}$/heart rate monitor (Nonin Medical Inc., Plymouth, MN, USA). The sensor was placed on the pregnant ewes’ teat and measurements were continuously recorded using LabChart 7 (ADInstruments, Bella Vista, NSW, Australia) (Darby et al., 2019; Duan et al., 2019). The maternal arterial, fetal femoral artery and amniotic catheters were connected to displacement transducers, a quad‐bridge amplifier and a data acquisition unit (PowerLab, ADInstruments) to record both maternal and fetal (corrected for amniotic pressure) blood pressure. All data were sampled at a rate of 1000 Hz, digitized and recorded using LabChart 7. The resulting blood pressure signals acted as real‐time external cardiac triggers for maternal and fetal MRI scanning (Duan et al., 2019, 2017; Schrauben et al., 2019).

Imaging was performed on a 3‐T clinical MRI system (MAGNETOM Skyra, Siemens Healthineers, Erlangen, Germany). MRI measurements were taken during three acquisition windows: a basal state (∼60 min) and then at two periods (TAD 1, ∼15–65 min; TAD 2, ∼75–135 min) post maternal intravenous tadalafil administration (24 mg, bolus, Sigma‐Aldrich).

### 2.4 Determination of blood flow within the maternal and fetal circulations

Either the maternal or the fetal arterial pressure waveform was used to generate a cardiac trigger for MRI of the maternal and fetal circulations, respectively (Duan et al., 2019; Schrauben et al., 2019). Two‐dimensional cine PC imaging was performed to measure blood flow within the maternal and fetal circulations with corresponding vessel appropriate velocity encoding (VENC). Maternal PC‐MRI acquisitions were completed for the ascending aorta (AAo; 150 cm/s) and the left and right uterine arteries (150 cm/s). Fetal blood flow was determined in the umbilical vein (UV; 100 cm/s). The following parameters were used: flip angle: 30°; repetition time: 7 ms; echo time: 3.18 ms; field of view: 240 mm; in‐plane resolution: 1.0 × 1.0 mm^2^; slice thickness: 5.0 mm; number of signal averages: 3; views per segment: 2 – according to our previously published technique (Cho et al., 2020; Dimasi et al., 2021; Duan et al., 2019; Saini et al., 2021). With ∼15 acquired phases in the cardiac cycle, these parameters achieve a temporal resolution of ∼28 ms. The typical acquisition time for each vessel was ∼2 min. PC cine images were acquired in the short‐axis plane of the vessels of interest, which were prescribed using two perpendicular long‐axis views of each vessel. Maternal left ventricular cardiac output (LVCO) was determined as equal to AAo blood flow and did not include coronary blood flow. UtA flow was determined as the sum of the left and right uterine arteries.

### 2.5 Determination of oxygen saturation

Due to the paramagnetic properties of deoxyhaemoglobin, the T_2_ relaxation time of blood is related to the oxygen saturation of blood (Christen et al., 2013). Vessel T_2_ oximetry was performed using a T_2_‐prepared pulse sequence with a balanced steady‐state free precession acquisition (Myomaps, Siemens) (Saini et al., 2020; Sun et al., 2015; Xu et al., 2020; Zhu et al., 2016). In‐plane resolution was 1.3 × 1.3 mm. MRI acquisition parameters over all subjects and vessels were: repetition time: 4.2 ms; echo time: 2.1 ms; flip angle: 70°; slice thickness: 6 mm; T_2_ preparation times: 32, 64, 96, 128, 160, 192 ms; and acquisition time: ∼50 s. A non‐rigid motion correction algorithm was applied to compensate for slight in‐plane fetal movement (co‐registration) (Giri et al., 2012). The T_2_ relaxation time for each vessel of interest was analysed using CVI^42^ (Circle Cardiovascular Imaging, Calgary, Canada). The regions of interest were manually adjusted for each image slice to cover the central 60% of the vessel of interest (UV, fetal DAo (descending aorta), left and right uterine veins) (Stainsby & Wright, 1998). Oxygen saturation was then calculated from T_2_ relaxation time using the T_2_‐oxygen saturation relationship for sheep blood as previously described (Saini et al., 2020).

### 2.6 Determination of oxygen delivery and consumption

Blood flow and T_2_‐derived oxygen saturations were combined to calculate overall fetal oxygen delivery ($D_{O_{2}}$), fetal oxygen consumption (${\dot{V}}_{O_{2}}$) and UtA $D_{O_{2}}$ using the following equations:

(1) $$\mathrm{Fetal}\;D_{O_{2}}=1.36\times \left[\mathrm{Hb}\right]\times Y_{\mathrm{UV}}\times {\dot{Q}}_{\mathrm{UV}}$$

(2) $$\mathrm{Fetal}\;{\dot{V}}_{O_{2}}=1.36\times \left[\mathrm{Hb}\right]\times \left(Y_{\mathrm{UV}}-Y_{\mathrm{DAo}}\right)\times {\dot{Q}}_{\mathrm{UV}}$$

(3) $$\mathrm{Uterine}\;\mathrm{artery}\;D_{O_{2}}=1.36\times \left[\mathrm{Hb}\right]\times Y_{\mathrm{UtA}}\times {\dot{Q}}_{\mathrm{UtA}}$$

${\dot{Q}}_{\mathrm{UV}}$ represents the measured umbilical vein blood flow; ${\dot{Q}}_{\mathrm{UtA}}$ represents the measured UtA blood flow; [Hb] represents either the mean fetal or maternal haemoglobin concentration during the relevant MRI acquisition window; 1.36 is the amount of oxygen (mL at one atmosphere) bound per gram of haemoglobin; *Y*_UV_ represents the oxygen saturation of UV blood; *Y*_DAo_ represents the oxygen saturation of the DAo blood, *Y*_UtA_ represents the oxygen saturation of UtA blood.

### 2.7 Placentome MRI model

Our previously published and validated sheep‐specific MRI signal model (Flouri et al., 2022, 2021) is of the form:

$$S\;\left(b,T_{E}\right)=S_{0}\;\left[e^{-bd*}\left(fe^{-T_{E}R_{2}^{f_{b}}}+ve^{-T_{E}R_{2}^{mb}}\right)+\left(1-f-v\right)e^{-bd-T_{E}R_{2}^{ts}}\right]$$

where S is the measured MR signal and $S_{0}$ is the signal with no diffusion weighting (i.e., *b* = 0). The five independent model parameters are the feto‐placental blood volume fraction f, trophoblast apparent diffusivity d, pseudo‐diffusivity $d^{*}$, feto‐placental blood relaxation $R_{2}^{f_{b}}=\;1/T_{2}^{f_{b}}$ and maternal blood volume fraction v. We used literature‐based values for highly saturated maternal blood relaxation $R_{2}^{mb}$ and tissue relaxation $R_{2}^{ts}$ at 3 T of 150 ms^−1^ and 42 ms^−1^ (de Bazelaire et al., 2004; Stanisz et al., 2005).

Notably this model estimates separate parameters of the fetal and maternal circulations to the placenta including the placentome fetal and maternal blood volume fractions, the tissue diffusivity, and the feto‐placental blood oxygen saturation. Hence this model allows an interrogation of the placental interaction between maternal and fetal responses to physiological conditions in the individuals.

### 2.8 Placentome image analysis and model fitting

Sheep placentomes were manually segmented using ITK‐SNAP (Version 3.6.0, 2017, www.itksnap.org). To reduce motion artefact, a rigid registration (Klein et al., 2010) was applied followed by a non‐rigid free‐form registration (Flouri et al., 2020). Voxel‐by‐voxel model fitting was performed with a Levenberg–Marquardt algorithm applied to the placentome MRI model using an in‐house software developed in MATLAB (The MathWorks, Natick, MA, USA). We previously applied the same model‐fitting approach (Flouri et al., 2022, 2021). The model fitting was initialized with parameter estimates from the model fitting results obtained from average placentome region of interest signal curves as in Melbourne et al. (2019).

To stabilize the fitting the following constraints were chosen: 0 < f < 1 (no units), 0 < d < 1 (mm^2^ s^−1^), 0 < $d^{*}\;$< 1 (mm^2^ s^−1^), 0 < $T_{2}^{f_{b}}\;$< 150 and 0 < *𝑣 <* 1 (no units)

### 2.9 Blood sampling and fetal blood gas measurements

After fetal surgery, fetal arterial blood samples (0.5 mL) were collected daily to monitor fetal health by measuring the partial pressure of oxygen ($P_{aO_{2}}$), partial pressure of carbon dioxide ($P_{\mathrm{aC}O_{2}}$), oxygen saturation ($S_{aO_{2}}$), pH, haemoglobin, haematocrit, base excess and lactate concentrations, temperature corrected to 39°C for sheep blood with a RAPIDPOINT 500 (Siemens Healthineers, Melbourne, Australia). During the MRI scan, arterial blood samples (0.5 mL) for maternal and fetal blood gas analysis were taken at the beginning and end of each state and venous blood samples (3 mL) were collected prior to and then 15, 30, 75, 95 and 135 min post tadalafil administration for subsequent maternal and fetal tadalafil plasma concentration analysis.

### 2.10 Quantification of tadalafil plasma concentrations in the maternal and fetal circulations

Quantitation of plasma tadalafil concentrations was determined using liquid chromatography (LC; Shimadzu Nexera XR, Shimadzu, Japan) coupled to a SCIEX 4500 Triple‐Quad system (MS/MS; SCIEX, Framingham, MA, USA). In brief, 300 μL of acetonitrile containing 1 μg/mL tadalafil‐d3 (Cayman Chemical Co., Ann Arbor, MI, USA) was added to 100 μL plasma. Samples were vortexed for 1 min and then centrifuged at 16,000 *g* for 10 min at 4°C. Supernatant was injected on a 1.7 μM ACQUITY UPLC BEH C18 column (Waters Corporation, Milford, MA, USA). Mobile phases were 0.2% formic acid in water (A) and acetonitrile (B). Plasma concentrations were calculated using a standard curve that was prepared in blank maternal sheep plasma spiked with 5– 5000 ng/mL tadalafil (Sigma‐Aldrich).

### 2.11 Post‐mortem

At 123–124 dGA pregnant ewes were humanely killed with an overdose of sodium pentobarbitone (Virbac, Peakhurst, NSW, Australia) and the fetus was delivered via hysterotomy and weighed (3.160 ± 0.130 kg; mean ± SD).

### 2.12 Statistical analysis

To determine the effect of tadalafil on blood flow, oxygen delivery, oxygen consumption and PP, a repeated measures one‐way ANOVA was used with Bonferroni's correction for multiple comparisons (GraphPad Prism version 8, GraphPad Software, San Diego, CA, USA). The impact of tadalafil on maternal/fetal blood pressure and heart rate was determined using repeated measures one‐way ANOVA with time (analysed as 5 min averages every 5 min) as the repeated measure. Data are presented as means ± SD and a probability of 5% (*P *< 0.05) was considered significant for all analyses.

## 3 RESULTS

### 3.1 Maternal and fetal plasma tadalafil concentrations, blood gas, pH, Hb, Hct and lactate measures

**FIGURE 1:**
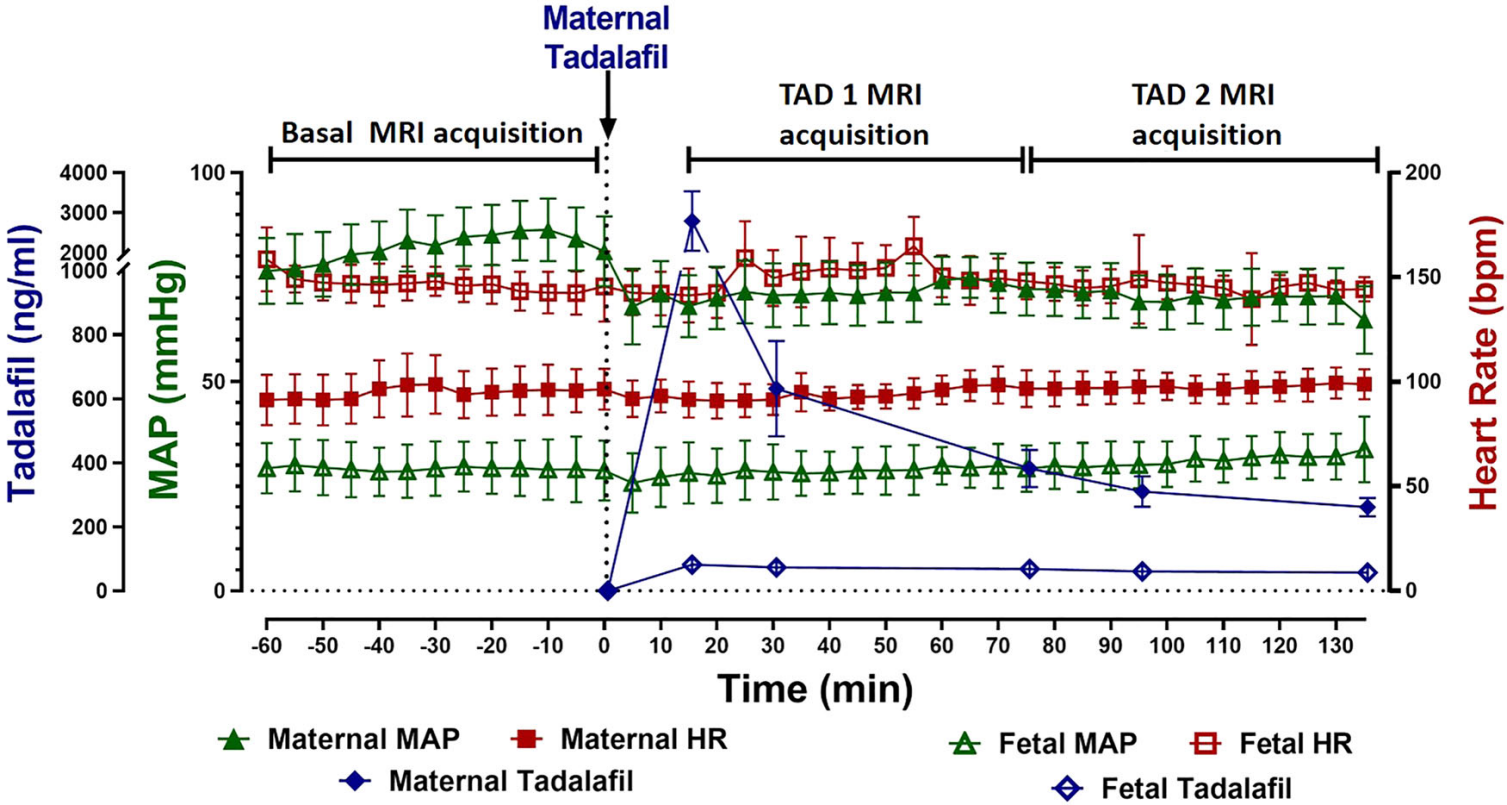
Maternal (filled symbols) and fetal (open symbols) MAP (triangles), HR (squares) and plasma tadalafil concentrations (diamonds) across the basal, TAD 1 and TAD 2 MRI acquisition periods. Maternal MAP is significantly lower during both TAD 1 and TAD 2 periods in comparison to baseline. Data analysed by repeated measures one‐way ANOVA with a Bonferroni correction for multiple comparisons. Data presented as means ± SD. *P* ≤ 0.05.

Following maternal tadalafil administration, maternal and fetal plasma concentrations both peaked at 15 min (Figure 1). Mean maternal tadalafil concentrations were higher during the TAD 1 period than the TAD 2 period (*P* = 0.016; Table 1). After the initial peak in plasma tadalafil concentrations, the fetal:maternal plasma tadalafil ratio ranged from 0.13 to 0.22 (0.19 ± 0.04; mean ± SD).

**TABLE 1:** Maternal and fetal blood gases, Hb and lactate values prior to anaesthesia for MRI and during Basal and TAD periods.

	Pre‐anaesthesia (*n* = 7)	Basal (*n* = 7)	TAD 1 (15–65 min post‐tadalafil) (*n* = 7)	TAD 2 (75–135 min post‐tadalafil) (*n* = 7)	*P*
Maternal venous parameters					
Tadalafil (ng/mL)	—	—	1270 ± 740	318 ± 108	*0.016*
$P_{O_{2}}$ (mmHg)	40.8 ± 7.1 ^a^	64.3 ± 9.0 ^b^	67.2 ± 9.9 ^b^	73.5 ± 7.5 ^b^	*<0.0001*
$P_{CO_{2}}$ (mmHg)	40.6 ± 4.3	41.7 ± 5.3	45.2 ± 7.4	45.2 ± 7.1	0.191
pH	7.444 ± 0.024 ^a^	7.387 ± 0.038 ^b^	7.374 ± 0.044 ^b^	7.372 ± 0.041 ^b^	*0.035*
$S_{O_{2}}$ (%)	68.4 ± 8.1 ^a^	87.2 ± 2.5 ^b^	87.8 ± 4.4 ^b^	90.6 ± 3.2 ^b^	*0.002*
Hb (g/L)	111.7 ± 7.5 ^a^	80.2 ± 8.0 ^b^	83.1 ± 7.4 ^b^	83.3 ± 8.5 ^b^	*<0.0001*
Hct (%)	33 ± 2 ^a^	24 ± 2 ^b^	25 ± 2 ^b^	25 ± 3 ^b^	*<0.0001*
Lactate (mmol/L)	1.01 ± 0.66	0.88 ± 0.29	0.88 ± 0.23	0.86 ± 0.20	0.635
Fetal arterial parameters					
Tadalafil (ng/mL)	—	—	74 ± 16	62 ± 21	ns
$P_{aO_{2}}$ (mmHg)	19.5 ± 1.9	20.9 ± 2.9	19.0 ± 3.3	18.7 ± 3.1	0.198
$P_{CO_{2}}$ (mmHg)	45.9 ± 4.7 ^a^	56.1 ± 6.3 ^b^	61.2 ± 7.1 ^c^	63.8 ± 5.8 ^c^	*0.002*
pH	7.392 ± 0.025 ^a^	7.308 ± 0.037 ^b^	7.293 ± 0.025 ^b^	7.292 ± 0.023 ^b^	*0.001*
$S_{aO_{2}}$ (%)	65.9 ± 5.0	62.4 ± 5.6	54.8 ± 10.0	53.9 ± 10.0	0.125
Hb (g/L)	104.5 ± 8.6	94.7 ± 6.3	98.9 ± 5.6	98.4 ± 5.6	0.160
Hct (%)	29 ± 2	28 ± 2	29 ± 2	29 ± 2	0.153
Lactate (mmol/L)	1.40 ± 0.11 ^a^	1.67 ± 0.30 ^a^	2.08 ± 0.39 ^b^	2.32 ± 0.39 ^b^	*0.001*

*Note*: Values are means ± SD. Data analysed by repeated measures one‐way ANOVA with Bonferroni's correction for multiple comparisons. ns, *P *> 0.05. Superscript letters indicate significant differences between time points (*P *< 0.05) such that values with different letters are statistically different from each other and values with the same letter are not different. The difference between plasma tadalafil concentrations in the TAD 1 and TAD 2 period was determined by a paired *t*‐test. *P*‐values shown in italic are significant (*P* < 0.05).

Maternal $P_{O_{2}}$ (*P *< 0.0001) and $S_{O_{2}}$ (*P* = 0.002) were significantly higher during all MRI periods than before anaesthesia (*P *< 0.0001); however, neither $P_{O_{2}}$ nor $S_{O_{2}}$ was different between basal, TAD 1 or TAD 2 periods (Table 1). Maternal pH (*P* = 0.035), haemoglobin (*P *< 0.0001) and haematocrit (*P *< 0.0001) were significantly lower during all MRI periods than before anaesthesia but were not different between basal, TAD 1 or TAD 2 periods (Table 1). There was no difference in maternal $P_{CO_{2}}$ (*P* = 0.191) or lactate (*P* = 0.635) across all periods. Fetal $P_{aO_{2}}$ (*P* = 0.1975), $S_{aO_{2}}$ (*P* = 0.1253), haemoglobin (*P* = 0.1596) and haematocrit (*P* = 0.153) remained stable from before anaesthesia throughout the experimental protocol (Table 1). Fetal $P_{\mathrm{aC}O_{2}}$ (*P* = 0.002) and lactate (*P* = 0.001) were significantly higher during all MRI periods than pre‐anaesthesia. Both fetal $P_{\mathrm{aC}O_{2}}$ and fetal lactate were significantly increased during the TAD 1 and TAD 2 periods compared to the basal period. Fetal pH was significantly lower during all MRI periods than pre‐anaesthesia, but was not different between basal, TAD 1 or TAD 2 periods (*P* = 0.001; Table 1).

### 3.2 Effect of TAD on maternal and fetal blood pressure and heart rate

Maternal tadalafil treatment significantly reduced maternal MAP for the duration of both TAD 1 and TAD 2 periods (*P* = 0.001; Figure 1). There was no impact of maternal TAD on maternal HR, fetal HR or fetal MAP (Figure 1).

### 3.3 Impact of tadalafil on maternal LVCO, UtA blood flow and uterine $D_{O_{2}}$

**FIGURE 2:**
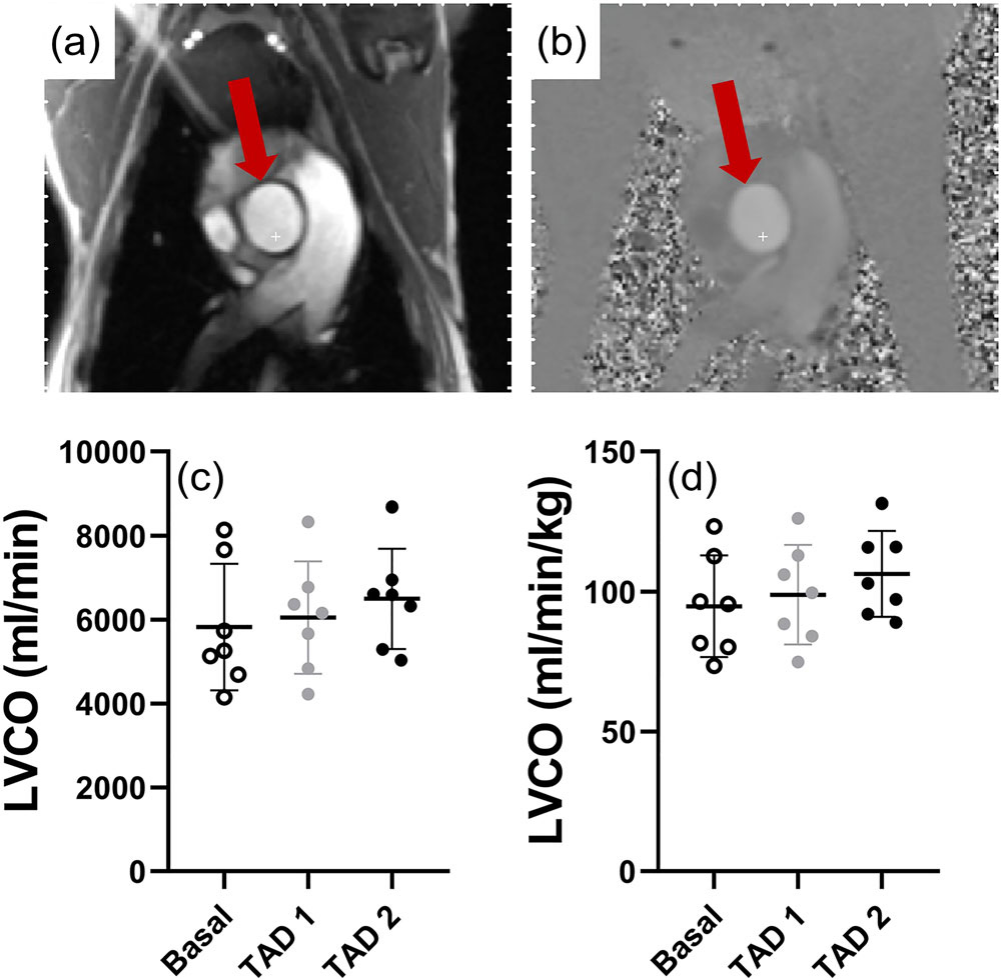
Representative anatomical‐magnitude (a) and phase (b) images of the maternal ascending aorta utilized for the determination of maternal left ventricular cardiac output (LVCO) unindexed (c) and indexed (d) to maternal weight across basal (open circles), TAD 1 (grey filled circles) and TAD 2 (black filled circles) periods. Data presented as individual data points with means ± SD superimposed. Data analysed by a repeated measures one‐way ANOVA with Bonferroni's correction for multiple comparisons.

**FIGURE 3:**
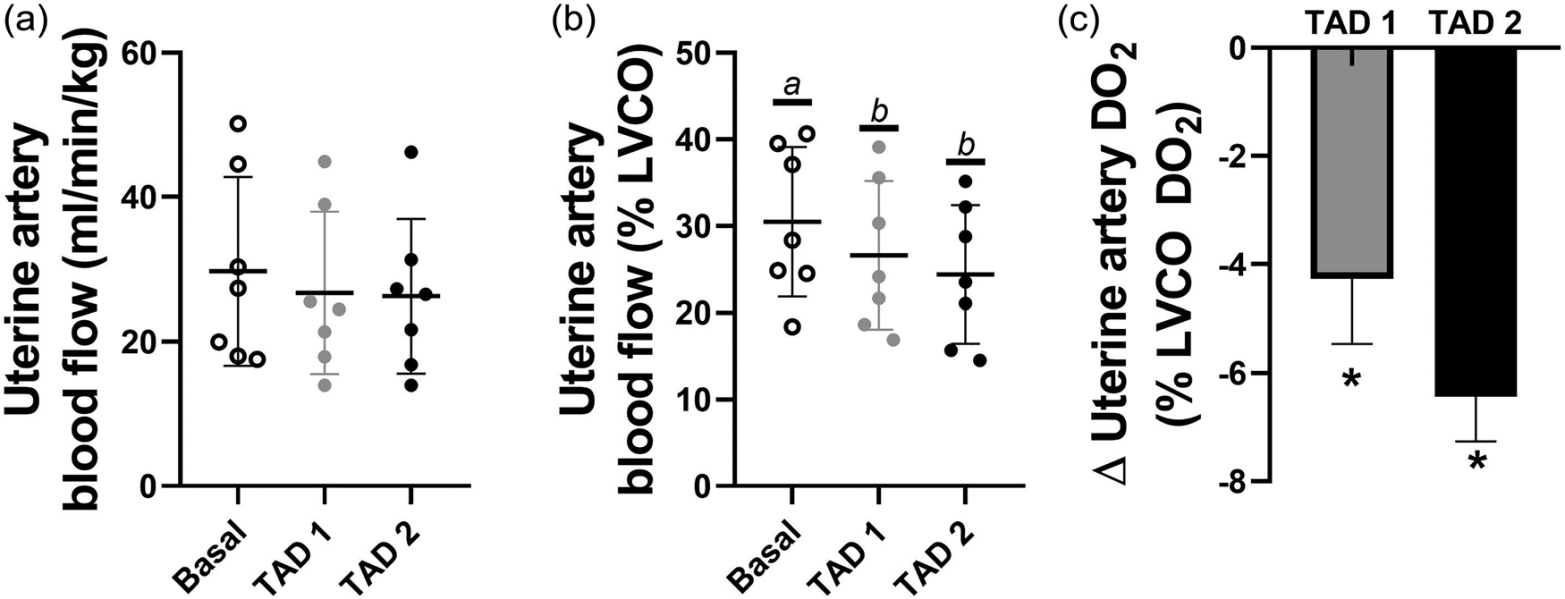
Uterine artery blood flow indexed to maternal weight (a), normalized to LVCO (b) and the change from basal state of uterine artery $D_{O_{2}}$ as a proportion of available $D_{O_{2}}$ (c). Data presented as individual data points with means ± SD superimposed. Data analysed by a repeated measures one‐way ANOVA with Bonferroni's correction for multiple comparisons. Letters represent statistical differences between groups whereby different letters depict statistical significance; *statistically different from baseline *P* ≤ 0.05.

Maternal tadalafil administration did not change maternal LVCO from baseline during either the TAD 1 or TAD 2 periods (*P* = 0.075; Figure 2c,d). There were also no changes in absolute UtA blood flow (*P* = 0.093). However, maternal tadalafil administration significantly decreased UtA blood flow distribution such that the proportion of LVCO blood flow directed toward the uterus via the uterine arteries was lower during the TAD 1 and TAD 2 periods than during baseline (*P* = 0.001; Figure 3b). This resulted in a decrease in available oxygen delivery to the uterus in both the TAD 1 and TAD 2 periods (*P* = 0.037; Figure 3c). There were no significant relationships between maternal tadalafil concentrations and either UtA blood flow or UtA $D_{O_{2}}$ (data not shown).

### 3.4 Effect of tadalafil on the placenta

**FIGURE 4:**
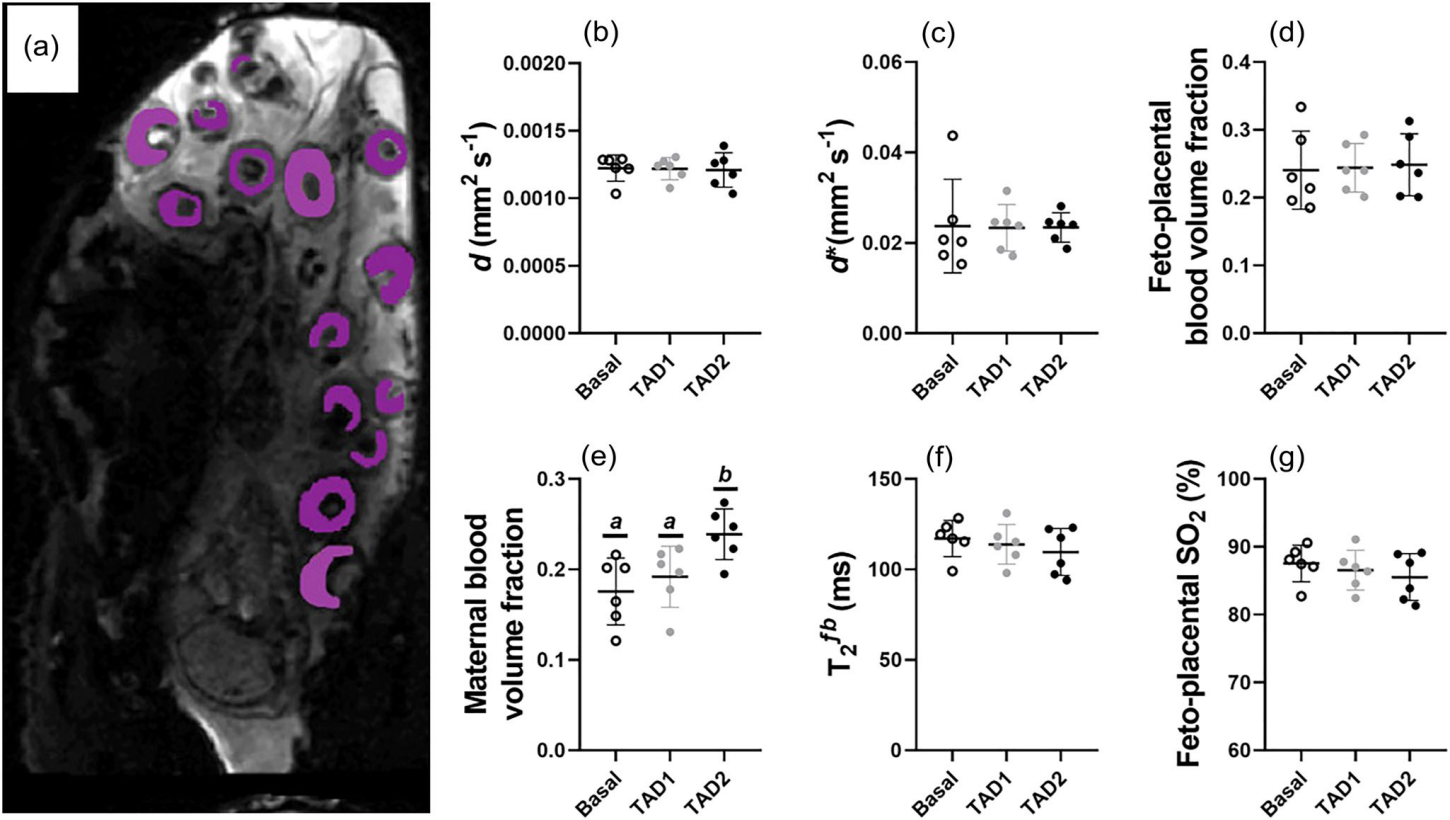
Representative placentome image showing regions of interest for placentome DECIDE (a) and the corresponding diffusivity (b), pseudo‐diffusivity (c), placentome fetal blood volume fraction (d), placentome maternal blood volume fraction (e), T_2_ of the placentome fetal blood fraction (f) and converted feto‐placental $S_{O_{2}}$ (g) during basal, TAD 1 and TAD 2 periods. Data presented as individual data points with means ± SD superimposed. Data analysed by a repeated measures one‐way ANOVA with Bonferroni's correction for multiple comparisons. Letters represent statistical differences between groups whereby different letters depict statistical significance; *statistically different from baseline *P* ≤ 0.05.

Maternal tadalafil administration had no impact on placentome diffusivity (*P* = 0.866; Figure 4b), pseudo‐diffusivity (Figure 4c), feto‐placental blood volume fraction (*P* = 0.817; Figure 4d), T_2_ of fetal blood within the placentomes (*P* = 0.135; Figure 4f) or feto‐placentome $S_{O_{2}}$ (*P* = 0.125; Figure 4g). Maternal tadalafil administration significantly increased the fraction of maternal blood within placentomes during the TAD 2 period (*P* = 0.004; Figure 4e). There were no significant relationships between maternal tadalafil concentrations and placentome diffusivity, pseudo‐diffusivity, placentome fetal blood volume fraction, placentome maternal blood volume fraction, T_2_ of fetal blood within the placentomes or feto‐placentome $S_{O_{2}}$ (data not shown).

### 3.5 Effect of tadalafil on fetal oxygen delivery and consumption

**FIGURE 5:**
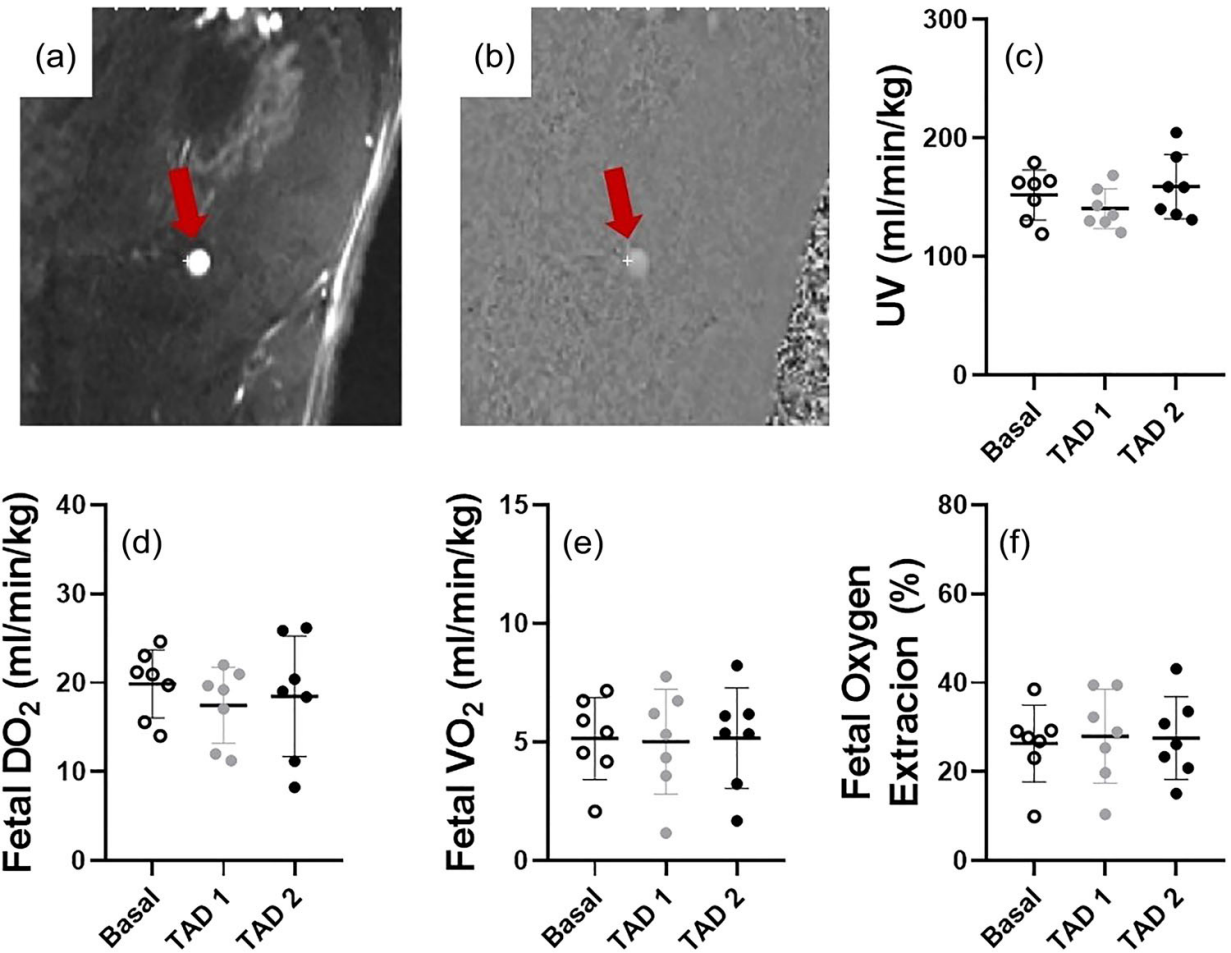
Representative anatomical magnitude (a) with corresponding phase (b) image of the umbilical vein and umbilical vein blood flow (c), fetal $D_{O_{2}}$ (d), fetal ${\dot{V}}_{O_{2}}$ (e) and fetal oxygen extraction fraction (f) during the basal, TAD 1 and TAD 2 periods. Data presented as individual data points with means ± SD superimposed. Data analysed by a repeated measures one‐way ANOVA with Bonferroni's correction for multiple comparisons. Letters represent statistical differences between groups whereby different letters depict statistical significance; *statistically different from baseline *P* ≤ 0.05.

Maternal tadalafil treatment did not impact UV blood flow (*P* = 0.188; Figure 5c), fetal $D_{O_{2}}$ (*P* = 0.437; Figure 5d), fetal ${\dot{V}}_{O_{2}}$ (*P* = 0.930; Figure 5e) or fetal oxygen extraction fraction (*P* = 0.852; Figure 5f).

## 4 DISCUSSION

Herein we utilized a combination of clinically relevant advanced MRI techniques to determine the impact of tadalafil on maternal haemodynamics, PP and both fetal oxygen delivery and consumption. We found that maternal tadalafil treatment reduced UtA blood flow as a proportion of maternal cardiac output. At the level of the placenta, apart from an increase in maternal blood volume fraction, there was no impact of tadalafil, and this corresponded to no change in fetal oxygen delivery or consumption.

The well‐characterized ability of PDE_5_ inhibitors such as tadalafil to increase blood flow by way of arterial vasodilatation in the setting of pulmonary hypertension has led to the hypothesis that these same PDE_5_ inhibitors may also be useful in placental insufficiency‐induced FGR by increasing maternal blood flow to the uterus (Rotella, 2002). Several preclinical studies have suggested the benefit of sildenafil in treating FGR in rodent and sheep models (Herraiz et al., 2012; Satterfield et al., 2010). This was supported by a small observational case–control study of oral sildenafil (25 mg three times a day), which suggested a potential benefit of the drug in early‐onset FGR (von Dadelszen et al., 2011). Conversely and prior to the onset of the STRIDER trials, intravenous sildenafil (100 mg bolus+4 mg/h infusion) was found to decrease UtA blood flow in pregnant ewes carrying growth‐restricted fetuses (Miller et al., 2009), resulting in impaired fetal oxygenation, hypotension and tachycardia. The negative results of the STRIDER trials in relation to pregnancy outcomes in early‐onset FGR, and the safety concerns in neonates whose mothers received sildenafil led the consortium to advise that clinicians should stop prescribing sildenafil in cases of FGR (Groom et al., 2018). Although data on UtA blood flow are not available from STRIDER participants, in keeping with the negative results it has been reported that sildenafil did not alter the concentrations of circulating angiogenic factors (Sharp et al., 2018). Certainly, the differences in outcomes associated with sildenafil exposure within preclinical studies and compared to human studies have been postulated to be due to an ineffective sildenafil dose. Specifically, a meta‐analysis found that the most effective dose for improving fetal growth and maternal blood pressure regulation would be an order of magnitude higher than that administered to STRIDER participants (Paauw et al., 2017). In addition, the negative results could be due to the short half‐life of sildenafil, prompting exploration of tadalafil with its longer half‐life as a more viable and stable intervention.

In the present study, we found that like sildenafil (Miller et al., 2009), tadalafil also significantly decreased the proportion of total cardiac output to the UtA in the pregnant ewe. This contrasts with the limited work in humans (Tanaka et al., 2019) where higher blood concentrations of tadalafil across five pregnant women affected by either FGR or preeclampsia were associated with higher UtA blood flows (Tanaka et al., 2019). One possible explanation for the differing results in humans and sheep could be the route of tadalafil delivery in that Tanaka *et al.* administered tadalafil as an oral formulation leading to a slower release into the maternal circulation, resulting in lower peak concentrations (∼300–400 ng/mL). In contrast, we administered tadalafil intravenously, leading to mean maternal tadalafil concentrations being approximately 3–4 times higher (∼1200 ng/mL; Table 1) during the TAD 1 period but concentrations in the TAD 2 period that were quite similar (∼300 ng/mL; Table 1) to those previously reported (Tanaka et al., 2019). This is important because even during the TAD 2 period where similar tadalafil concentrations were associated with higher UtA blood flow in pregnant women and despite the persistent decrease in maternal MAP over both TAD periods, we found no impact of tadalafil on absolute UtA blood flow.

We employed DECIDE imaging to investigate the direct or indirect impact that maternal tadalafil treatment may have had on the placenta. Parameters associated with PP and diffusivity were not different during either TAD period with the exception of an increase in the maternal blood volume fraction. Interestingly, tadalafil treatment has previously been shown to decrease maternal blood pressure and dilate the placental maternal blood sinuses in a mouse model of preeclampsia (Yoshikawa et al., 2017). Given that we found tadalafil to have a similar impact on maternal blood pressure, albeit without initial prevailing hypertension, we may have identified a conserved response to tadalafil across species. That being said, it is not clear whether the increase in maternal blood volume fraction is due to either a direct effect of tadalafil dilating the placental maternal blood sinuses or an indirect response to a decrease in placental maternal flow pressure and flow rate. The latter would imply that there is a compensating effect in the placenta in response to the reduced proportion of UtA blood flow in an attempt to aid in the maintenance of fetal oxygen extraction.

A strength of the present study was our ability to directly measure fetal blood pressure and heart rate as well as to obtain fetal blood samples for blood gas and tadalafil concentration analysis. Tadalafil concentrations in human fetuses are ∼1/4 of maternal tadalafil concentrations whereas our data suggest that tadalafil concentrations in fetal sheep are ∼1/5 of maternal concentrations (Kubo, Tanaka et al., 2017). This suggests that tadalafil crosses the sheep placenta to a fairly similar extent as it does in humans. Indeed, the tadalafil concentrations (mean ∼60–75 ng/mL) in fetal sheep were in line with those previously reported in cord blood during a phase 1 clinical trial (∼40 ng/mL, range = 3.9–141 ng/mL; Kubo, Tanaka et al., 2017). As such, fetal sheep were directly exposed to clinically relevant tadalafil concentrations. Despite this direct tadalafil exposure, we found that fetal blood pressure and heart rate during the TAD 1 and TAD 2 periods were not different from baseline. Furthermore, tadalafil had no impact on the MRI‐derived measures of fetal oxygen delivery and consumption. Whilst an ideal intervention for FGR would not directly influence fetal haemodynamics, it should be noted that fetuses in the present study were normoxic and normally grown. Further studies utilizing a sheep model of FGR would be required to understand whether these same tadalafil concentrations would have a direct impact on a circulatory system that has already adapted to a lower substrate supply (e.g., brain‐sparing physiology).

Establishing the effectiveness of tadalafil treatment for pregnancies complicated by FGR is the current focus of the TADAFER11b trial (Maki et al., 2022), a trial that was approved following promising results derived from an FGR cohort that did not complete full recruitment (Maki et al., 2019). Herein, we have shown that during a relatively short exposure period, maternal tadalafil treatment does not increase absolute UtA blood flow in an uncomplicated sheep model of human pregnancy. Further preclinical studies are needed to better understand whether tadalafil may have a different impact during a pregnancy complicated by FGR and/or when a more prolonged tadalafil treatment period is assessed. Clinical studies of acute effects of tadalafil on circulation (Nii et al., 2023) should be performed prior to introduction of longer‐term studies. Previously, we and others have shown that sildenafil directly increases pulmonary blood flow and decreases right to left heart shunting through the foramen ovale in fetal sheep (De Bie et al., 2021; Morrison et al., 2022). This finding may have been predictive of the increased rates of persistent pulmonary hypertension during the Dutch arm of the STRIDER trials (Pels et al., 2020). Although we did not assess the entirety of fetal circulation in this study, it is paramount that future studies perform a more comprehensive assessment of fetal haemodynamics in response to tadalafil exposure.

## AUTHOR CONTRIBUTIONS

Conception or design of the work: Jack R. T. Darby, Christopher K. Macgowan, Mike Seed, Andrew Melbourne, Janna L. Morrison. Acquisition or analysis or interpretation of data for the work: Jack R. T. Darby, Dimitra Flouri, Georgia K. Williams, Steven K. S. Cho, Ashley S. Meakin, Stacey L. Holman, Michael D. Wiese, Christopher K. Macgowan, Mike Seed, Andrew Melbourne, Janna L. Morrison Drafting the work or revising it critically for important intellectual content: Jack R. T. Darby, Dimitra Flouri, Stacey L. Holman, Christopher K. Macgowan, Mike Seed, Anna L. David, Andrew Melbourne, Janna L. Morrison. All authors have read and approved the final version of this manuscript and agree to be accountable for all aspects of the work in ensuring that questions related to the accuracy or integrity of any part of the work are appropriately investigated and resolved. All persons designated as authors qualify for authorship, and all those who qualify for authorship are listed.

## CONFLICT OF INTEREST

The authors declare no conflicts of interest.

## Data Availability

Data supporting the findings of this manuscript are available from the Figshare online repository: https://doi.org/10.6084/m9.figshare.25427362.
